# Costs and consequences of treatment for mild gestational diabetes mellitus – evaluation from the ACHOIS randomised trial

**DOI:** 10.1186/1471-2393-7-27

**Published:** 2007-10-28

**Authors:** John R Moss, Caroline A Crowther, Janet E Hiller, Kristyn J Willson, Jeffrey S Robinson, The Australian Carbohydrate Intolerance Study in Pregnant Women (ACHOIS) Trial Group

**Affiliations:** 1 Discipline of Public Health, The University of Adelaide, Adelaide, South Australia, 5005, Australia; 2 Discipline of Obstetrics and Gynaecology, The University of Adelaide, Women's and Children's Hospital, King William Road, North Adelaide, South Australia, 5006, Australia

## Abstract

**Background:**

Recommended best practice is that economic evaluation of health care interventions should be integral with randomised clinical trials. We performed a cost-consequence analysis of treating women with mild gestational diabetes mellitus by dietary advice, blood glucose monitoring and insulin therapy as needed compared with routine pregnancy care, using patient-level data from a multi-centre randomised clinical trial.

**Methods:**

Women with a singleton pregnancy who had mild gestational diabetes diagnosed by an oral glucose-tolerance test between 24 and 34 weeks' gestation and their infants were included. Clinical outcomes and outpatient costs derived from all women and infants in the trial. Inpatient costs derived from women and infants attending the hospital contributing the largest number of enrolments (26.1%), and charges to women and their families derived from a subsample of participants from that hospital (in 2002 Australian dollars). Occasions of service and health outcomes were adjusted for maternal age, ethnicity and parity. Analysis of variance was used with bootstrapping to confirm results. Primary clinical outcomes were serious perinatal complications; admission to neonatal nursery; jaundice requiring phototherapy; induction of labour and caesarean delivery. Economic outcome measures were outpatient and inpatient costs, and charges to women and their families.

**Results:**

For every 100 women with a singleton pregnancy and positive oral glucose tolerance test who were offered treatment for mild gestational diabetes mellitus in addition to routine obstetric care, $53,985 additional direct costs were incurred at the obstetric hospital, $6,521 additional charges were incurred by women and their families, 9.7 additional women experienced induction of labour, and 8.6 more babies were admitted to a neonatal nursery. However, 2.2 fewer babies experienced serious perinatal complication and 1.0 fewer babies experienced perinatal death. The incremental cost per additional serious perinatal complication prevented was $27,503, per perinatal death prevented was $60,506 and per discounted life-year gained was $2,988.

**Conclusion:**

It is likely that the general public in high-income countries such as Australia would find reductions in perinatal mortality and in serious perinatal complications sufficient to justify additional health service and personal monetary charges. Over the whole lifespan, the incremental cost per extra life-year gained is highly favourable.

**Trial Registration:**

Australian Clinical Trials Registry ACTRN12606000294550

## Background

Since resources are inescapably scarce, health care interventions should be assessed for their impact on costs as well as on clinical outcomes. Recommended best practice is that economic evaluation should be integral with randomised clinical trials [1]. This has the advantage of generating patient-level data for a relatively modest research protocol cost.

The background, methods and pregnancy outcomes of the Australian Carbohydrate Intolerance Study in Pregnant Women (ACHOIS) trial have been reported elsewhere [2]. Briefly, a multi-centre randomised clinical trial was conducted from 1993 to 2003 to determine whether treatment of women with mild gestational diabetes mellitus reduced the risk of serious perinatal complications. Women between 24 and 34 weeks' gestation who had mild gestational diabetes were randomly assigned to receive dietary advice, blood glucose monitoring, and insulin therapy as needed (Intervention Group) or routine care according to standard practice at each centre. (Routine-Care Group).

Eligible women had a singleton or twin pregnancy between 16 and 30 weeks' gestation, attended antenatal clinics at the collaborating hospitals, and after screening positive for risk of gestational diabetes had a 75 g oral glucose-tolerance (OGTT) test at 24 to 34 weeks' gestation in which the venous plasma glucose level was less than 7.8 mmol/L after an overnight fast and 7.8 to 11.0 mmol/L at two hours [3]. Women with more severe glucose impairment were not eligible for the ACHOIS trial.

## Methods

The economic evaluation took the perspective of the health system and its patients and compared the direct costs of the additional resources used from randomisation until the time of postnatal hospital discharge of the woman or her baby, whichever occurred last, with the increments in health outcome for both the woman and the baby in a cost-consequences analysis [4,5]. Women with a twin pregnancy were excluded from this economic evaluation because it was anticipated that their likely increased use of resources could not be robustly estimated due to their expected small numbers in the trial. This analysis included all costs, but only those primary clinical outcomes (consequences) that achieved a P-value less than 0.05 in the main trial after adjustment for the confounders of maternal age, race or ethnic group, and parity. The economic evaluation was designed to take the form of a cost-consequences analysis, since trading-off between the utilities (preference values) of mother and infant would entail difficult conceptual and ethical issues. The intention was to provide relevant information about the incremental value of the intervention to assist decision-makers in setting priorities for health resource allocation.

The costing reported in this paper began at the moment of randomisation, and ended at the initial postnatal hospital discharge of the woman or her baby, whichever occurred last. Direct costs were measured to the health system and charges to the woman and her family. Cost was calculated as the number of occasions of each service multiplied by its unit cost. More than 98% of participants were public hospital patients.

Obstetric hospital outpatient occasions of service after enrolment were collected from the trial data for all women with a singleton pregnancy. Unit costs were obtained from sources congruent with the Manual of Resource Items for use in Major Submissions to the Australian Pharmaceutical Benefits Advisory Committee involving Economic Analyses [6]. The Manual recognises sources that include the Schedule of Medicare Benefits [7], the Pharmaceutical Benefits Schedule [8], and the Department of Veterans' Affairs Schedule of Fees [9]. Unit costs of relevant services are presented in Table 1.

**Table 1:** Unit costs of health services associated with the ambulatory management of gestational diabetes mellitus.

**Item**	**Unit cost (Australian dollars 2002)**	**Source**
Antenatal clinic visits	$28.75	MBS item 16500
Physician clinic visits:		
initial	$119.35	MBS items 110, 116 (consultant physician)
subsequent	$59.75	
Dietician visits:		DVA
initial	$63.84	DT-03 ($5.32*12)
subsequent	$31.92	DT-22 ($5.32*6)
Diabetes educator	$31.92	As for dietician subsequent visit (see above)
Insulin therapy	$46.26 per week	Weighted average DPMQ for 11 PBS insulin items; Mean duration 6.4 weeks Intervention Group, 5.4 weeks Routine-Care Group

MBS = Medicare Benefits Schedule 1 November 2001 including 1 May 2002 Supplement; DVA = Department of Veterans' Affairs schedule of fees for services in a public hospital, 2002; PBS = Schedule of Pharmaceutical Benefits, November 2001; weighted by Health Insurance Commission data on overall population use; DPMQ = Dispensed Price for Maximum Quantity.

Inpatient costing was only able to be performed for women and babies who were born at the Women's and Children's Hospital (WCH), Adelaide, Australia; the hospital which recruited the largest number of women (261/1,000; 26.1%) and which is typical of a large metropolitan tertiary care centre in Australia. The participating clinicians in the ACHOIS multi-centre trial had agreed to a common clinical management protocol. The trial began before computerised inpatient cost information systems became routinely available in the participating hospitals. From 1995–96 onwards, inpatient costs were obtained from Trendstar^® ^(McDonnell Douglas Information Systems), the WCH patient costing system, based on input from several feeder systems, including nursing dependency levels. In this system, inpatient separations are case mix-classified according to Australian-Refined Diagnosis Related Groups. Cost weights come from the National Hospital Cost Data Collection [10] and include overheads. Inpatient costs for the baby were added to those of the mother.

Charges to women and their families were obtained by a questionnaire survey of a sample of 108 South Australian study participants (majority from the WCH) after funding for this became available, from January 1997 to June 2003. The themes covered in the questionnaire included time off work and costs for food, hospital parking, childcare, and blood sugar monitoring equipment. Data from general practitioners or community service providers were not collected because of research budgetary constraints. Costs were expressed in calendar year 2002 Australian dollars, adjusted by the Consumer Price Index (CPI) [11]. Based on 2002 purchasing power parities, one Australian dollar would convert to 0.74 US dollars, 0.48 UK pounds or 0.66 Euros [12].

The statistical analysis of the main ACHOIS trial with a sample size of 1000 women has been described elsewhere [2]. A pragmatic judgment was made that the sample size for the economic evaluation should be determined by the limits of detection of differences in the primary outcome measures in the main trial. The power of the study to show differences in total health service costs was 7.5% and for patient changes 17.5%, assuming normality. As is common in health economic evaluation, the distribution of the costs per patient of managing gestational diabetes mellitus was skewed; hence bootstrapping (using 10000 resamples) was used to confirm the results of the analysis of variance [13]. All occasions of service and health outcomes were adjusted by maternal age, race or ethnic group, and parity.

## Results

### Health service use and direct outpatient costs for study participants

Of the 970 women in the ACHOIS trial with a singleton pregnancy, 474 were assigned to the Intervention Group and 496 to the Routine-Care Group. Women in the Intervention Group made 0.7 fewer antenatal clinic visits (p = 0.0002), but 2.5 more specialist medical clinic visits (p < 0.0001), 1.56 more dietician visits (p < 0.0001), 1.79 more diabetes educator visits (p < 0.0001), and received insulin therapy more often (adjusted RR 6.18, 95%CI 3.69 to 10.35, p < 0.0001) than women in the Routine-Care Group (Table 2). Thus, across these five types of hospital antenatal outpatient services, the mean direct costs were $337 greater for women in the Intervention Group compared with women in the Routine-Care Group (Table 3).

**Table 2:** Health services use after enrolment by women with a singleton gestation and their infants

	**Intervention group**	**Routine-care group**	**Adjusted treatment effect^# ^(95%CI)**	**Adjusted p-value**
	n = 474	n = 496		

**Antenatal services after enrolment**				
Antenatal inpatient admission	135 (28.5)	133(26.8)	1.11(0.91–1.36)	0.31
Antenatal clinic visits*	4.4 (1–7)	5.2(3–7)	-0.70(-1.06, -0.33)	0.0002
Specialist clinic visits*	4.0 (1–7)	1.3(0–2)	2.50(2.13, 2.87)	<0.0001
Dietician visits*	1.7 (1–2)	0.2(0–0)	1.56(1.39, 1.72)	<0.0001
Diabetes educator visits*	2.0 (1–2)	0.2(0–0)	1.79(1.59, 1.98)	<0.0001
Insulin therapy*	96 (20.3)	16 (3.2)	6.18(3.69–10.35)	<0.0001
**Birthing services**				
Induction of labour	183 (38.6)	148 (29.8)	1.34(1.13–1.60)	0.001
Caesarean birth	142(30.0)	153 (30.8)	0.97(0.80–1.17)	0.76
**Neonatal services**				
Paediatrician present at delivery	211 (44.5)	225 (45.4)	1.02 (0.89–1.17)	0.72
Admission to neonatal nursery	330 (69.6)	294 (59.3)	1.15 (1.04–1.26)	0.004
Hours of phototherapy^§^	45(24–72)	36(24–51)		0.27

Dichotomous outcomes reported as number (%) of women/babies with relative risk and 95% confidence intervals (CI) as treatment effect; *continuous outcomes reported as mean (interquartile range) with mean difference 95% CIs as treatment effect.

^§^Hours of phototherapy is median (interquartile range).

#Adjusted for maternal age, race or ethnic group and parity.

**Table 3:** Adjusted mean direct hospital outpatient costs for women with a singleton gestation

	**Intervention group**	**Routine-care group**	
	**(n = 474)**	**(n = 496)**	
	**Adjusted mean cost**	**Adjusted mean cost**	**Difference in adjusted mean cost**

Antenatal clinic visits	$125	$145	-$20
Specialist clinic visits	$327	$153	$174
Dietician visits	$87	$11	$76
Diabetes educator	$67	$10	$57
Insulin therapy	$69	$18	$51
Total	$674	$337	$337

Unit costs from Table 1. Occasions of service from main ACHOIS data base.

Adjusted for maternal age, race or ethnic group, and parity.

Rounded to whole dollars

### Primary clinical outcomes

Singleton infants in the Intervention Group were less likely to experience any serious perinatal outcome than infants in the Routine-Care Group (adjusted RR 0.33, 95%CI 0.14 to 0.76, p = 0.01), but were more likely to be admitted to a neonatal nursery (adjusted RR 1.15, 95%CI 1.04 to 1.26, p = 0.004) (Table 4). Women with a singleton pregnancy in the Intervention Group were more likely to have an induction of labour (adjusted RR = 1.34, 95%CI 1.13 to 1.60, p = 0.001), but were no more likely to have a caesarean delivery (Table 4).

**Table 4:** Primary clinical outcomes among women with a singleton gestation and their infants

	**Intervention group**	**Routine-care group**	**Adjusted RR (95% CI)**	**Adjusted p-value**
**Infants**	**n = 474**	**n = 496**		
Any serious perinatal complication	7 (1.5)	23 (4.6)	0.33 (0.14–0.76)	0.01
Death	0 (0.0)	5 (1.0)		0.07
Shoulder dystocia	7 (1.5)	16 (3.2)	0.46 (0.19–1.12)	0.09
Bone fracture	0 (0.0)	1 (0.2)		0.38
Nerve palsy	0 (0.0)	3 (0.6)		0.11
Admission to neonatal nursery	330 (69.6)	294 (59.3)	1.15 (1.04–1.26)	0.004
Jaundice requiring phototherapy	44 (9.3)	42 (8.5)	1.06 (0.71–1.59)	0.76
**Women**	**n = 474**	**n = 496**		
Induction of labour	183 (38.6)	148 (29.8)	1.34 (1.13–1.60)	0.001
Caesarean delivery	142 (30.0)	153 (30.8)	0.97 (0.80–1.17)	0.76

Values are number (%) of babies or women

Adjusted for maternal age, race or ethnic group, and parity

### Inpatient costs

The inpatient costing sample consisted of 195 participating women with a singleton pregnancy from the WCH. Compared to the remainder of the women with a singleton pregnancy in the ACHOIS trial, this sample was similar in maternal age and body mass index, but more likely to be nulliparous (p = 0.01), Caucasian (p = 0.01), and to be of lower socioeconomic status (p = 0.03). At trial entry these women had a higher OGTT fasting result (p = 0.005), but similar two hour OGTT result (p = 0.52), and had a five-day greater gestational age (p < 0.0001) compared to the remainder of women in the trial. The mean days of initial postnatal hospital stay for both these singleton women and their infants at the WCH, with adjustment for year of admission, were not significantly different from that for women in the other participating hospitals. At the WCH, the direct costs for inpatient services for women and infants (singleton) were $202 greater in the Intervention Group compared with women and infants in the Routine-Care Group, not a statistically significant difference (p = 0.84).

### Charges to participating women and their families

Of the 108 women with a singleton pregnancy providing data from the questionnaire survey, the mean adjusted charge from randomisation until birth, for women in the Intervention Group and their families, was $367 compared with $302 for the Routine-Care Group (p = 0.34) (Table 5).

**Table 5:** Mean charges* to women and their family from randomisation to birth.

	**Intervention group (n = 52)**	**Routine-care group (n = 56)**
	Adjusted mean	Adjusted mean
Paid child care	$24	$13
Travel (to & from appts.)	$46	$42
Blood glucose monitoring equipment & consumables	$53	$17
Food substitution: additional expenditure	$20	$10
Subtotal	$143	$82
Mother time off paid work	$84	$145
Partner time off work	$141	$75
Total	$367	$302

* In 2002 Australian dollars, pecuniary and non-pecuniary costs. Wages calculated at the rate of Average Weekly Earnings (ABS).

Adjusted for maternal age, race or ethnic group, and parity.

Rounded to whole dollars.

Costs did not differ between groups

### Costs and consequences of treatment for mild GDM

Over the course of the ACHOIS trial, for every 100 women with a singleton pregnancy and a positive OGTT who were offered treatment of mild gestational diabetes mellitus in addition to standard obstetric care (Table 6),

**Table 6:** Summary of direct costs post-randomisation and consequences of management of mild gestational diabetes mellitus expressed per 100 women with a singleton gestation

	**Intervention group (n = 100)**	**Routine-care group (n = 100)**	**Difference (n = 100)**
**Direct costs post-randomisation-**			
Antenatal ambulatory services *			
Antenatal clinic	$12,479	$14,483	$-2,004
Specialist clinic	$32,657	$15,290	$17,367
Dietician	$8,716	$1,127	$7,589
Diabetes educator	$6,692	$982	$5,710
Insulin therapy	$6,888	$1,800	$5,088
*Subtotal*	*$67,432*	*$33,681*	*$33,751*
**Inpatient services**			
Hospital costs†	$545,125	$524,891	$20,234
***Total direct health service costs***	***$612,557***	***$558,572***	***$53,985***
Patient/family costs‡	$36,749	$30,229	$6,521
**Consequences**§			
**Infants**			
Any serious perinatal complication¶	1.1	3.2	-2.2
Admission to neonatal nursery	68.1	59.5	8.6
**Women**			
Induction of labour	38.0	28.3	9.7

*Antenatal ambulatory services post-randomisation based on ACHOIS trial data for 970 women (474 in Intervention group and 496 in Routine care group respectively).

† Hospital costs for all mothers and babies based on WCH patient costing system (obtained from data for 195 inpatient women with a singleton pregnancy at the WCH).

‡ Patient/family costs obtained by survey of a patient subsample of 108 South Australian study participants.

§Based on ACHOIS trial data for 474 and 496 singleton women (respectively) (see Table 4).

¶Serious perinatal complications were defined prospectively as one or more of the following: death, shoulder dystocia, bone fracture, and nerve palsy.

Adjusted for maternal age, race or ethnic group, and parity.

▪ $53,985 additional direct costs were incurred at the obstetric hospital,

▪ $6,521 additional charges were incurred by the women and their families,

▪ 9.7 additional women experienced an induction of labour, and

▪ 8.6 more babies were admitted to a neonatal nursery, yet

▪ 2.2 fewer babies experienced any serious perinatal complication (includes perinatal death, shoulder dystocia, bone fracture, and nerve palsy), and

▪ 1.0 fewer babies experienced a perinatal death.

### Incremental cost-effectiveness ratio

Although a cost-consequences analysis was planned, the actual trial results enabled the estimation of a credible cost-effectiveness ratio in terms of the incremental cost for a reduction in any serious perinatal complication. The incremental cost per additional serious perinatal complication prevented (defined prospectively as one or more of the following: death, shoulder dystocia, bone fracture, and nerve palsy) was therefore $27,503 (= ($53,985 + $6,521)/2.2). The incremental cost per perinatal death prevented was $60,506 (= ($53,985 + $6,521)/1.0). Based on Australian life tables at the midpoint of the trial [14], and a discount rate of 5%, the incremental cost per life-year saved was $2,988 (=($53,985 + $6,521)/20.25).

## Discussion

The ACHOIS trial [2] has demonstrated the clinical effectiveness of active treatment of women with mild gestational diabetes and this paper reports the cost-effectiveness of such treatment. Although a cost-consequence analysis was planned, the actual trial data allowed a credible cost-effectiveness ratio to be estimated, demonstrating that the form of an economic analysis often cannot be settled until data on effectiveness and cost are actually available [15]. The ACHOIS trial also demonstrated that the health-related quality of life of the intervention mothers was better, both during the antenatal period and three months after birth [2].

Our economic analysis used primary clinical outcome information and use of hospital services for all 970 women with a singleton pregnancy recruited to the ACHOIS trial. For assessing inpatient resource use, the relative differences in costs incurred by women and infants at the hospital contributing the largest number of women (n = 195) were assumed to be representative of those across the whole study. This representation was used because the trial began before computerised inpatient cost information systems became routinely available in the participating hospitals.

The size of this study was limited by the sample size of 1000 women in the ACHOIS trial and the small sample of women able to be assessed for charges to families. The power of the study to show differences was therefore low. Even with these limitations however, these data are of importance as there have been no previous reports of costs of treatment options for women with gestational diabetes from randomised trials.

It is likely that the general public in high-income countries such as Australia would find the reduction in perinatal mortality sufficient to justify the additional costs of $60,506, whether or not society places a larger value on a baby's life than on that of other members of the general public. Even incremented to current year costs, the figures would remain highly favourable. Indeed, at a value of $2,988, the incremental cost per life-year gained is highly favourable. By way of comparison, George *et al*. [16] found that historically the Australian Pharmaceutical Benefits Advisory Committee was unlikely to recommend a drug for government subsidy if the additional cost per life-year gained exceeded $86,154 and was unlikely to reject a drug for which the additional cost per life-year gained was less than $47,612 (2002 Australian dollar values).

The results suggest that being diagnosed with mild gestational diabetes mellitus and receiving the recommended care is not associated with any significant increase in the direct inpatient costs or postnatal length of stay, but is associated with an increase in the women's antenatal outpatient costs. This includes costs due to an increase in the number of visits to a physician, dietician and diabetes educator. The economic evaluation has been confined to in-trial costs and consequences. No modelling has yet been attempted to account for either costs or consequences beyond the end of the trial.

## Conclusion

Treatment of women with gestational diabetes by dietary advice, blood glucose monitoring and insulin therapy as needed resulted in a reduction in serious perinatal complications, although more women experienced an induction of labour, and more of their infants were admitted to the neonatal nursery [2]. Treatment of women with a singleton pregnancy who have gestational diabetes in this way resulted in increased direct costs of outpatient, obstetric hospital services and direct out of pocket charges for women and families compared with routine pregnancy care. However, taking a perspective over the whole lifespan, the incremental cost per extra life-year gained is highly favourable. It is likely that the general public in high-income countries such as Australia would find the reductions in perinatal mortality and in serious perinatal complications sufficient to justify the additional health service and personal monetary costs.

## Competing interests

The author(s) declare that they have no competing interests.

## Authors' contributions

All authors contributed to the study design, interpretation of the data and preparation of the drafts of the manuscript. In addition CC coordinated the trial and the collection of data. KW performed the data analyses. All authors read and approved the final manuscript.

## Pre-publication history

The pre-publication history for this paper can be accessed here:


